# Comprehensive analysis of tandem amino acid repeats from ten angiosperm genomes

**DOI:** 10.1186/1471-2164-12-632

**Published:** 2011-12-23

**Authors:** Yuan Zhou, Jing Liu, Lei Han, Zhi-Gang Li, Ziding Zhang

**Affiliations:** 1 State Key Laboratory of Agrobiotechnology, College of Biological Sciences, China Agricultural University, Beijing 100193, China

## Abstract

**Background:**

The presence of tandem amino acid repeats (AARs) is one of the signatures of eukaryotic proteins. AARs were thought to be frequently involved in bio-molecular interactions. Comprehensive studies that primarily focused on metazoan AARs have suggested that AARs are evolving rapidly and are highly variable among species. However, there is still controversy over causal factors of this inter-species variation. In this work, we attempted to investigate this topic mainly by comparing AARs in orthologous proteins from ten angiosperm genomes.

**Results:**

Angiosperm AAR content is positively correlated with the GC content of the protein coding sequence. However, based on observations from fungal AARs and insect AARs, we argue that the applicability of this kind of correlation is limited by AAR residue composition and species' life history traits. Angiosperm AARs also tend to be fast evolving and structurally disordered, supporting the results of comprehensive analyses of metazoans. The functions of conserved long AARs are summarized. Finally, we propose that the rapid mRNA decay rate, alternative splicing and tissue specificity are regulatory processes that are associated with angiosperm proteins harboring AARs.

**Conclusions:**

Our investigation suggests that GC content is a predictor of AAR content in the protein coding sequence under certain conditions. Although angiosperm AARs lack conservation and 3D structure, a fraction of the proteins that contain AARs may be functionally important and are under extensive regulation in plant cells.

## Background

Tandem amino acid repeats (AARs), or homopeptides, are protein segments that comprise a continuous array of identical residues. As repetitive DNA is very abundant in eukaryotic genomes [1], AARs are frequently found in the proteomes of eukaryotes [2-4]. These simple peptides can be encoded by tandem repeats of the same codon, which are vulnerable to point mutations, or by a mixture of synonymous codons [5]. These repetitive codon tracts are primarily introduced by either replication slippage [6] or recombination [7].

AARs are often situated in disordered regions of proteins that lack regular 3D structures [8]. Nevertheless, over the past two decades, increasing attention has been paid on biological importance of AARs (see [9] for a recent review) which have long been regarded as junk sequences [10]. AARs have been shown to be associated with several diseases. For example, the expansion of a glutamine repeat may induce Huntington's disease and other neuro-degenerative diseases [11]. Beneficial effects of AARs have also been uncovered. An example is the glutamine repeat that appears in a key component of the biological clock in the fungus *Neurospora crassa *White Collar-1. This AAR was suggested to control circadian period length [12]. Large-scale analyses indicate that AARs tend to participate in the regulation of transcription [8,13,14] and are frequently involved in protein-protein interactions [15].

AARs are highly polymorphic and fast-evolving sequences [9,16]. In line with the accelerated rate of evolution for protein segments that are in or near AARs, selective constraints are thought to be relaxed around AARs [8,17]. There are competing interpretations of the rapid evolution of AARs. Some believed that AARs evolve in a largely neutral fashion [18], partly as a consequence of the balance between replication slippage and point mutations [6]. Based on shifts of the frequency distribution of coding tri-nucleotide repeats compared to that of non-coding tri-nucleotide repeats, Mularoni *et al. *proposed that selection plays an important role in AAR evolution [19]. There is also evidence for positive selection on the AARs from case studies of a few mammalian genes [20,21].

The frequency and size of AARs show inter- and intra-species variation both in large-scale comparisons [17] and in studies focused on vertebrates [8,13,19] or fruit flies [22]. The causal factors underlying this variation are still a matter of dispute [13,17], and some have attributed them to GC content bias [16,18,23]. In plants, repetitive DNA is widely used as a genetic marker, and its variation among transcripts has been observed [24]. Nevertheless, in contrast to animal AARs, plant AARs have not been intensively investigated, except in a recent report based on two model plants, Arabidopsis (*Arabidopsis thaliana*) and rice (*Oryza sativa*) [14].

In this report, we revisit the questions surrounding AARs in plants in the light of current accumulation of plant whole genome sequences. A comparison of 1-to-1 orthologous proteins between ten sequenced angiosperm species revealed a positive correlation between AAR content and GC content, a finding that may be applicable to some other non-metazoan taxa. Other factors related to AAR content variation were also discussed. We attempted to summarize the functions of conserved long angiosperm AARs and their host genes in the context of the rapid evolution of AARs and their flanking regions. Our analysis also supports the idea that AARs are widely associated with protein structural disorder. Finally, we suggest that transcripts of repeat-containing proteins (RCPs) are under various levels of regulation in plant cells.

## Results and Discussion

### Correlation between AAR content and GC content in plants

We predefined an AAR as an uninterrupted run of four or more identical amino acids. Our 1-to-1ortholog dataset contains 4, 281 groups of proteins from six eudicots[25][26][27][28][29][30]* (A. thaliana*, *Carica papaya*, *Glycine max*, *Malus × domestica*, *Populus trichocarpa *and *Vitis vinifera*) and four monocots [31],[32][33][34] (*Brachypodium distachyon*, *O. sativa*, *Sorghum bicolor *and *Zea mays*). The abbreviations used and general information about the genome sequence data of these ten species are given in Table 1. All of the following analyses within the spectrum of angiosperm were based on this dataset, unless stated otherwise.

**Table 1:** Summary of the ten angiosperm genomes included in this study

Organism	Abbreviation	Genome size (Mbp)	Number of proteins	Reference
**Eudicot**				
*Arabidopsis thaliana*	Arabidopsis	120	27, 1692	25
*Carica papaya*	papaya	372	27, 181	[26]
*Glycine max*	soybean	1, 100	46, 260	[27]
*Malus × domestica*	apple	742	62, 997	[28]
*Populus trichocarpa*	cottonwood	550	40, 664	[29]
*Vitis vinifera*	grape	500	26, 092	[30]
**Monocot**				
*Brachypodium distachyon*	false brome	272	25, 525	[32]
*Oryza sativa*	rice	382	56, 795	[31]
*Sorghum bicolor*	sorghum	735	27, 561	[33]
*Zea mays*	maize	2, 500	32, 606	[34]

The genome size is the total length of chromosomal sequences. A gene with multiple protein products was counted as one protein in this table.

Similar to other eukaryotes, angiosperm proteins are enriched in AARs (0.84 AAR per protein on average). Because short AARs may be derived from the interruption of a long AAR, we used repeated residues per 1000 amino acids (RRPK, Repeated Residues per Kilo Amino Acids, defined as the ratio of the total AAR length to the protein length, multiplied by 1000) to represent the AAR content of a protein or protein segment. For example, the RRPK of peptide "QQQQQSTWQQQQAAE" is 9/15 × 1000 = 600. There is a nearly 3-fold variation in RRPK between these ten species, with values that range from 5.06 (grape) to 15.25 (rice). It is somehow striking that large genomes or proteomes do not necessarily have higher RRPK.

**Figure 1:**
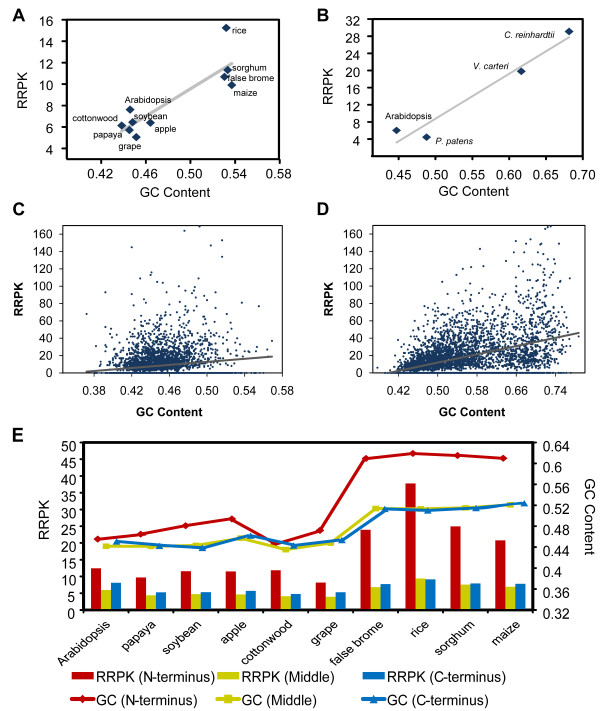
**Correlation between AAR and coding GC content in plants**. (A) Among angiosperm orthologs (the abbreviations used are detailed in Table 1), the average values are shown; (B) among Arabidopsis, moss and green algae orthologs, the average values are shown; (C) for all angiosperm orthologs from Arabidopsis; (D) for all angiosperm orthologs from rice; (E) in different regions of orthologous angiosperm proteins.

We noticed that orthologs in monocots have an elevated RRPK and that the GC content (of their coding sequences) is also higher. A strong linear correlation between RRPK and GC content can be observed (Pearson's correlation coefficient, r = 0.87, p = 1.1 × 10^-3^; Figure 1A), although no positive correlations observed within eudicots or monocots, partially due to a limited taxonomy coverage of the available genomes. To test whether this phenomenon is specific to angiosperms, we also tested a set of 1-to-1 orthologs between Arabidopsis, moss (*Physcomitrella patens *[35]) and green algae (*Chlamydomonas reinhardtii *[36] and *Volvox carteri *[37]) and also observed a strong positive correlation (r = 0.97, p = 2.5 × 10^-2^; Figure 1B). Moreover, within each angiosperm species, a weak but significant positive correlation between protein RRPK and GC content was observed (r = 0.15~0.43, p < 1.0 × 10^-10^; Figure 1C and 1D). Finally, when protein sequences were equally divided into three parts, an accumulation of AARs in the N-terminus could be observed, which is in line with the elevated GC content of this region (Figure 1E). However, the C-terminus also had a higher RRPK in comparison with the middle segments for all of the species except rice (Figure 1E), similar to what has been observed in animals [16,22]. It is worth mentioning that AARs have been proposed to follow a negative gradient from the N-terminus to the C-terminus in plant proteins [14]. One explanation for this discrepancy is that there are differences in the definition of the protein terminus between the two studies. Zhang et al. measured the absolute position of the AARs [14] and thus may have counted all of the repeats that are present in short proteins as being in the N-terminus.

High GC content favors replication slippage and, thus, the generation of AARs, which has been proposed in a number of reports [13,16,38]. On the other hand, GC content has also been treated as an indicator of the local recombination rate [39]. Angiosperm species with high recombination rates (i.e., exactly, higher average centiMorgan per megabase), such as Arabidopsis and rice [40], are relatively enriched for AARs (Figure 1A). We attempted to test the association of RCPs with recombination hotspots in Arabidopsis by exploiting publicly available extensive SNP data [41]. A total of 293 putative hotspot neighboring genes (see Materials and Methods) were identified. At the whole proteome level, the fraction of hotspot neighboring genes in genes encoding RCPs is higher than that in genes not encoding RCPs in our dataset (1.3% vs. 0.93%, Fisher's exact test, p = 0.01), indicating that the influence of recombination on the AAR frequency, although limited, cannot be excluded.

### The relationship between AAR content and GC content is distorted by AAR composition and life history traits

There is controversy over the relationship between GC content and AAR content. A positive correlation between GC content and AAR content has been observed in some mammalian species [16], while in a wider spectrum of taxa, a negative correlation was proposed [18]. Thus, we examined the relationship between GC content and AAR content within two additional taxonomic groups from distinct eukaryotic clades, *Sordariomycetes *fungi and *Diptera *insects (summarized in Table S1 and Table S2 in Additional File 1, respectively). These 1-to-1 ortholog groups contain 4, 047 and 3, 680 proteins from fungi and insects, respectively.

**Figure 2:**
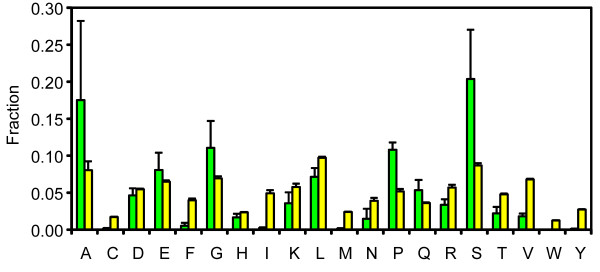
**Residue composition in AARs and across the entire set of orthologs**. The fractions of residues from AARs and from the entire set of orthologs are shown as green columns and yellow columns, respectively. Only positive error bars are shown.

A positive correlation was observed in fungi (r = 0.78, p = 2.2 × 10^-2^; Figure S1 in Additional File 1). Both plants and fungi harbor a large fraction of AARs that are encoded by GC-rich codons (39.3% and 36.9% on average, respectively; see also Figure 2), including alanine, glycine and proline repeats. Removal of these three types of AAR would diminish the positive correlation (p = 0.19 and 3.6 × 10^-2 ^for plants and fungi, respectively). In contrast, fruit flies, whose RRPK shows a negative correlation with GC content (r = -0.72, p = 8.9 × 10^-3^, Figure S2 in Additional File 1), were relatively enriched for glutamine repeats (22.1% on average) but not for types of AAR that were encoded by GC-rich codons (31.7% on average), indicating the influence of AAR composition on the relationship between AAR content and GC content. Additionally, no significant correlation was identified in the insect group as a whole because mosquito proteins, which contain fewer glutamine repeats (15.9% on average) than their fruit fly orthologs, seem to accumulate AARs with elevated GC content (Figure S2).

However, GC content and residue composition are not the only factors that influence the AAR content. For example, both maize and grape have relatively higher GC content (Figure 1A) and fewer glutamine repeats (< 4.5%), but their RRPK are the lowest among eudicots and monocots, respectively (Figure 1A). These two species share at least two life-history traits: (1) relatively "large body size" and (2) cross-pollination. We intentionally used quotes in this paragraph to emphasize that, owing to the high plasticity of plant development, caution should be used when linking body size to genomic signatures. Conversely, self-pollinating "small grasses", such as Arabidopsis and rice, have abundant AARs. Rice orthologs are so abundant in AARs that they appear as an outlier in the linear regression (Grubbs's test, p = 0.015; Figure 1A). A recent survey [24] showed that barley (*Hordeum vulgare*) has a higher fraction of RCPs than sugarcane (*Saccharum officinarum*). Interestingly, the former is a self-pollinating "small grass", whereas the latter is a cross-pollinating "large grass". In all, life-history traits are seeming cofactors of AAR content and deserve re-examination when more angiosperm genome sequences become available.

Taken together, the driving force shaping the presence and content of different types of AARs or low-complexity sequences appear to be complex, as was recently suggested for *Plasmodium falciparum *[42]. The interplay between GC content, AAR residue composition and life-history remains complicated and needs further investigation.

### Rapid evolution of angiosperm AARs and their functions

Like their animal counterparts [8], many angiosperm AARs have not been conserved over a long period of evolution. This trend is indicated by the observation that approximately 75% of AARs fail to align to the corresponding region in any of the other orthologs (i.e., the corresponding regions in the multiple alignment of other orthologs are filled with gaps). A faster rate of evolution, as estimated by average dN/dS ratio of the AAR flanking regions in comparison to RCPs as a whole, was also observed (Mann-Whitney U test, p < 1 × 10^-9 ^for all species; Table S3 in Additional File 2), supporting previous work that was conducted in other species [17,43]. Although it has been suggested that purifying selection is relaxed in flanking regions of AARs, only about 3% of these flanking regions show signs of positive selection, i.e., a dN/dS greater than 1 (Table S4 in Additional File 2). Assuming that the fraction of regions under positive selection would be underestimated by the average dN/dS, we also calculated pairwise dN/dS for three pairs of species: (1) Arabidopsis and papaya, (2) rice and false brome and (3) maize and sorghum. The fractions were still limited (Welch's t-test, p > 0.05; Table S4), indicating that positive selection is not a ubiquitous evolutionary process in AAR flanking regions.

Some pathogenesis-related proteins from *P. falciparum *were enriched with long asparagines repeats, and this phenomenon has been proposed as a reflection of selection against human immune systems [44]. We tested the function enrichment of rice RCPs with higher (> 20) RRPK, but did not find specific terms other than the regulation of transcription (detailed data not shown). Even though some molecular functions are enriched for RCPs [14], a loss of conservation can make the detailed genetic or biochemistry assays of the function of the AARs difficult. Fortunately, a few conserved long AARs still exist and can serve as targets for further experimental analysis. We chose conserved long AARs based on two criteria: (1) longer than seven residues and (2) the corresponding regions from at least eight other orthologs could be aligned with a multiple sequence alignment identity that is not lower than 50%. We then mapped these 18 AARs onto the corresponding regions in the Arabidopsis orthologs (Table 2 and Table S5 in Additional File 2) and found a few with indirect evidence [45][46][47][48][49] of being functionally important in Arabidopsis (Table 2). For example, truncation of the N-terminal acid domains containing long serine repeats from ABI3 (ABA INSENSITIVE 3) largely abolishes its activity [45]. Similarly, truncation of a domain with a glutamine rich region in SEU (SEUSS) can cause severe developmental defects [46]. Nonetheless, the functions of most of these 18 AARs and even the functions of their host genes remain to be surveyed (Table S5).

**Table 2:** Putative functions for conserved long AARs in Arabidopsis

Protein	Type	Description (gene/AAR)	Clue
AT3G24650.1	S	*ABI3 *is a key component of the ABA signal transduction pathway./See text for details of AAR function.	Biochemical [45]
AT1G43850.1	Q	*SEU*, together with *LUG *(*LEUNIG*), controls the development of several organs./See text for details of AAR function.	Phenotypic [46]
AT4G32551.1	Q	*LUG*, see above./The AAR is thought to be involved in the assembly of transcriptional co-repressors.	Speculation only [47]
AT5G67470.1	P	*FH6 *(*FORMIN HOMOLOG 6*) binds profilin and is involved in actin-nucleating activity./The AAR may directly contribute to its binding activity.	Speculation only [48]
AT1G25540.1	Q	*PFT1 *(*PHYTOCHROME AND FLOWERING TIME 1*) is a transcription factor that controls the flowering time./The AAR may be involved in transcriptional activation.	Speculation only [49]

### Angiosperm AARs tend to be structurally disordered and regulated at the transcript level

One explanation for the rapid evolution of AARs is that AARs tend to be disordered and thus lack structural constraints during evolution [50], an idea recently stressed by Simon and Hancock [8]. Indeed, disorder-promoting residues [51] such as serine, alanine, glutamine, glycine and proline are overrepresented in angiosperm AARs in comparison with the entire set of orthologs (Mann-Whitney U test, p < 0.01; Figure 2). To test whether angiosperm AARs tend to be fully disordered (i.e., embedded in disordered regions), we used PONDR^® ^VSL2B [52] and IUPred [53] to predict disordered regions in our set of orthologs. These two software packages take advantage of distinct features and strategies to predict ordered/disordered status for each residue. PONDR^® ^VSL2B and IUPRED disagree about the absolute fraction of fully disordered AARs (Table 3). In an independent benchmarking test with default cutoffs [54], IUPRED achieved 59.5% sensitivity with 95.6% specificity; the sensitivity of VSL2 reached 75.5%, but its specificity was 79.4% (the sensitivity of VSL2B was shown to be approximately 5% lower compared with VSL2 at a similar specificity [52]). Thus, this disagreement reflects the different trade-offs between false positives and false negatives that are inherent to different predictors. We tested whether the tendency of AARs to be fully disordered was significant by counting how many times, in 1000 trials, randomly selected equal length segments in RCPs showed a higher fraction of fully disordered segments. None of the 1000 random trials in any of the 10 species resulted in a higher fraction of fully disordered segments, suggesting that the fraction of fully disordered AARs is indeed significantly high (i.e., p < 0.001).

**Table 3:** Fraction of fully disordered AARs

Abbreviation	VSL2B Fraction	IUPred Fraction
Arabidopsis	82.5%	48.0%
papaya	77.8%	41.6%
soybean	78.6%	46.3%
apple	67.9%	45.0%
cottonwood	79.6%	45.5%
grape	73.5%	39.7%
false brome	80.2%	44.6%
rice	81.5%	45.6%
sorghum	80.2%	44.9%
maize	78.4%	42.4%

All fractions of fully disordered AARs were significantly high in comparison with random samples (p < 0.001).

**Figure 3:**
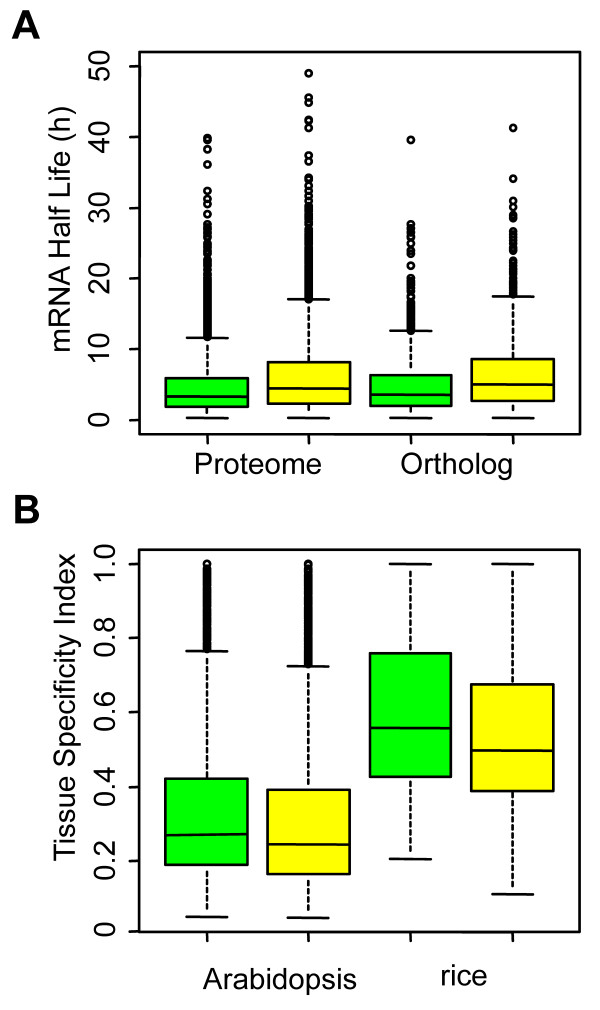
**Regulation of RCPs at the transcript level**. (A) Comparison of mRNA half lives of RCPs (green boxes) and non-RCPs (yellow boxes) in Arabidopsis. Large outliers (> 50 h) were not shown. (B) Comparison of the tissue specificity index of RCPs with a single gene model (green boxes) and non-RCPs with a single gene model (yellow boxes) in Arabidopsis and rice orthologs.

The tendency of AARs to be disordered indicates that RCPs may be under extensive regulation in plant cells, to prevent them from inducing dosage-sensitive phenotypes, as protein structural disorder was suggested to be associated with dosage-sensitive phenotypes in model metazoans [55]. The first line of evidence comes from the observation that transcripts encoding RCPs decay more quickly in Arabidopsis, similar to the transcripts of disordered proteins in human [56]. Both within orthologs and at the whole proteome scale, transcripts of RCPs have shorter half-lives than the rest (Welch's t-test, p < 2.2 × 10^-16^; Figure 3A). A similar result was obtained by comparing RCPs with non-RCPs encoded by GC-rich (GC content not smaller than 0.45) coding sequences (Welch's t-test, p < 2.2 × 10^-16^). Second, in the whole proteomes of Arabidopsis and rice, the fractions of alternatively spliced genes (i.e., genes with multiple gene models) were higher for RCPs than for non-RCPs (17.7% vs. 15.9% and 12.1% vs. 10.9%, respectively; Fisher exact test, p = 0.021 and 7.4 × 10^-3^, respectively). Moreover, protein segments encoded by alternatively spliced exons showed significantly higher repeat content than those encoded by constitutively spliced exons (Welch's t-test, p = 1.1 × 10^-28 ^and 5.1 × 10^-47 ^for Arabidopsis and rice, respectively; Table S6 in Additional File 2), which further supports the association between AARs and alternative splicing that has been proposed by Haerty and Golding based on their observations in metazoans [57]. For Arabidopsis orthologous RCPs and rice orthologous RCPs, a higher average RRPK of protein segments encoded by alternatively spliced exons was also observed (Welch's t-test, p = 7.6 × 10^-6 ^and 4.6 × 10^-11^, respectively; Table S7 in Additional File 2), while we found no higher fraction of RCPs to be alternatively spliced genes in comparison with non-RCPs (23.2% vs. 24.3% and 31.3% vs.37.7%, respectively; Fisher exact test, p = 0.31 and 7.7 × 10^-3^, respectively). A third regulatory process proposed here is tissue-specific expression, as measured by the tissue specificity index (see Materials and Methods), may be another complementary regulatory process. Orthologous RCPs from both Arabidopsis and rice had a relatively higher tissue specificity index than non-RCPs (Welch's t-test, p = 2.0 × 10^-4 ^and 1.4 × 10^-15^, respectively). If only genes with a single model were considered, the difference seemed to be limited but could still be observed (Welch's t-test, p = 1.5 × 10^-2 ^and 5.9 × 10^-11^, respectively), indicating that tissue-specific expression may partially complement alternative splicing in the regulation of RCPs.

## Conclusions

Angiosperm proteins are enriched in AARs whose content is positively correlated with the GC content in the coding sequences. It has also been suggested that the correlation between AAR content and GC content is influenced by residue composition of AARs as well as life-history traits. Similarly to AARs in many sequenced eukaryotic species, angiosperm AARs evolve rapidly and tend to be disordered. Although AARs are usually not well conserved, we identified 18 conserved long AARs for further detailed analysis. As potentially promiscuous molecules, RCPs are under at least three putative transcript-level regulatory controls in plant cells, including faster transcript decay, alternative splicing and tissue specificity of gene expression.

## Methods

### Collection of sequences

Sequences of *A. thaliana *(Version 9) and *O. sativa *(Version 6.1) were downloaded from TAIR [58] and RGAP [31], respectively. All of the other plant sequences were downloaded from the Phytozome 6.0 database [59]. The sources of the non-plant genome sequences are summarized in Tables S1 and S2 in Additional File 1. For genes with multiple protein products (gene models), only the representative one (if available) or the longest one was retained.

To search for 1-to-1 orthologs between species within certain taxonomic groups, InParanoid 4.1, one of algorithms with the lowest false-positive rates [60], was initially employed to identify pair-wise orthologs between the reference proteomes (*A. thaliana *for plant species, *N. crassa *for fungus species and *Drosophila melanogaster *for insects, excluding proteins encoded by the mitochondrial/chloroplast genomes) and the proteomes of the other species, with a score cutoff of 40. Sets of 1-to-1 orthologs found in all of the species within each group were obtained by collecting the intersection of the ortholog pairs.

### AAR identification, GC content calculation and statistical tests

We used in-house PERL scripts to collect data on the length, composition and position of AARs in protein sequences and to calculate the GC content. All statistical tests were implemented in R 2.12.1 [61].

### Recombination hotspots

We deduced the recombination hotspot at the Arabidopsis genome from SNP data described in [41]. Informative SNP markers in a chromosome were selected by the TAGGER application in HaploView 4.1 [62] with "-maxDistance 20 -aggressiveTagging -tagrsqcutoff 0.8" options, excluding SNPs identified as "N" in more than three out of 20 Arabidopsis accessions. Mainly due to the greedy marker selection approach of TAGGER along the whole chromosomes, the total number of informative SNP markers selected here is 60, 904. The hotspots were searched in 40-marker-long sliding windows by PHASE 2.1.1 [63], with "-MR1 1 -X10" options. These windows moved 20 markers per step. Windows that were longer than 100 kb were discarded. A hotspot was defined as a two-marker interval with a Bayes Factor that was higher than 10 in comparison with the background recombination rate [41]. Positions of the hotspot were compared with the position of genes in the Version 8 genome to collect genes that overlap with recombination hotspots. We called these genes putative hotspot neighboring genes (gene IDs were transferred to Version 9 for obsolete loci). We do not use Version 9 genomes here because approximately half of the markers fail to map to this version of genome [58].

### dN/dS calculation

The alignment of orthologous coding sequences was guided by a multi-protein sequence alignment that was generated by MAFFT 6.849 [64]. A flanking region was defined as 33 amino acids on both sides of an AAR; this region was truncated if the end of a protein was reached or if there was an adjacent AAR closer within 33 amino acids. PAML 4.3 yn00 tool [65] was used to calculate the dN/dS ratio with default parameters.

### Disordered region prediction

PONDR^® ^VSL2B [52] and IUPred [53] were used for predictions of disorder, with default parameters. We did not use PONDR^® ^VSL2 because of limitations in computational capability. We used default thresholds (0.5) to predict disordered residues for both predictors.

### Probing transcript level regulation features of RCPs

AtGenExpress [66] data (Accession: GSE5630, GSE5631, GSE5632, GSE5633 and GSE5634) were downloaded from the GEO database [67]. Data not derived from the Columbia-0 ecotype were discarded. A developmental time series of rice transcriptome data [68] (Accession: GSE13988, GSE14298, GSE14299 and GSE14300) was also downloaded from GEO [67]. Probes were mapped to loci in Arabidopsis and rice according to the mapping files that were provided by TAIR [58] and the Rice Array Database [69], respectively. All of the expression values that were presented above background (labeled as "Presence") were used and log-transformed. The expression values were normalized by subtracting the average expression value of a tissue and then adding the average expression value of the whole dataset. The tissue specificity index [70,71] was calculated as follows:

$$\text{Tissue}\;\text{Specificity}\;\text{Index}=\frac{\sum \left(1-\frac{{\text{X}}_{\text{i}}}{{\text{X}}_{\text{max}}}\right)}{\text{N}-1}$$

where x_i _is the expression value in the i^th ^tissue, x_max _is the highest expression value among all of the tissues and N is the total number of tissues. For loci with multiple probes, the average tissue specificity index was used.

To calculate the RRPK of the protein segments that were encoded by different types of exons, the protein sequences of RCPs were mapped to their exon sequences using our in-house PERL scripts. Only exons that encode proteins were retained for calculation. The mRNA half-life for each probe was obtained from [72]. For loci with multiple probes, the average mRNA half-life was used.

## Abbreviations

AAR: (Tandem) Amino Acid Repeat; RCP: Repeat Containing Protein; RRPK: Repeated Residues per One Kilo Amino Acids; SNP: Single Nucleotide Polymorphism.

## Authors' contributions

ZZ, JL and YZ conceived the study. JL and YZ performed most of the analyses. LH and ZGL helped perform the disorder prediction and the dN/dS calculation, respectively. YZ drafted the manuscript. ZZ supervised the study and revised the manuscript. All of the authors read and approved the final manuscript.

## Supplementary Material

Additional file 1 **The relationship between AAR content and GC content in fungi and insects**. This file contains two tables (Tables S1 and S2) that list the sources of the fungi and insect genome sequences that were used in this study, as well as two figures (Figures S1 and S2) that show the corresponding relationship between AAR content and GC content. Click here for file

Additional file 2 **Fast evolving AAR flanking regions, conserved long AARs of unknown function and RRPK of alternatively spliced exons**. This file contains Tables S3-S7. Click here for file

## References

[B1] Toth G Gaspari Z Jurka J Microsatellites in different eukaryotic genomes: survey and analysis Genome Res 2000 10 7 967 981 10.1101/gr.10.7.967 10899146 PMC310925

[B2] Green H Wang N Codon reiteration and the evolution of proteins Proc Natl Acad Sci USA 1994 91 10 4298 4302 10.1073/pnas.91.10.4298 8183904 PMC43772

[B3] Golding GB Simple sequence is abundant in eukaryotic proteins Protein Sci 1999 8 6 1358 1361 10.1110/ps.8.6.1358 10386886 PMC2144344

[B4] Karlin S Brocchieri L Bergman A Mrazek J Gentles AJ Amino acid runs in eukaryotic proteomes and disease associations Proc Natl Acad Sci USA 2002 99 1 333 338 10.1073/pnas.012608599 11782551 PMC117561

[B5] Alba MM Santibanez-Koref MF Hancock JM The comparative genomics of polyglutamine repeats: extreme differences in the codon organization of repeat-encoding regions between mammals and Drosophila J Mol Evol 2001 52 3 249 259 11428462 10.1007/s002390010153

[B6] Kruglyak S Durrett RT Schug MD Aquadro CF Equilibrium distributions of microsatellite repeat length resulting from a balance between slippage events and point mutations Proc Natl Acad Sci USA 1998 95 18 10774 10778 10.1073/pnas.95.18.10774 9724780 PMC27971

[B7] Richard GF Paques F Mini- and microsatellite expansions: the recombination connection EMBO Rep 2000 1 2 122 126 10.1093/embo-reports/kvd031 11265750 PMC1084263

[B8] Simon M Hancock JM Tandem and cryptic amino acid repeats accumulate in disordered regions of proteins Genome Biol 2009 10 6 R59 10.1186/gb-2009-10-6-r59 19486509 PMC2718493

[B9] Gemayel R Vinces MD Legendre M Verstrepen KJ Variable tandem repeats accelerate evolution of coding and regulatory sequences Annu Rev Genet 2010 44 445 477 10.1146/annurev-genet-072610-155046 20809801

[B10] Lovell SC Are non-functional, unfolded proteins ('junk proteins') common in the genome? FEBS Lett 2003 554 3 237 239 10.1016/S0014-5793(03)01223-7 14623072

[B11] Orr HT Zoghbi HY Trinucleotide repeat disorders Annu Rev Neurosci 2007 30 575 621 10.1146/annurev.neuro.29.051605.113042 17417937

[B12] Michael TP Park S Kim TS Booth J Byer A Sun Q Chory J Lee K Simple sequence repeats provide a substrate for phenotypic variation in the *Neurospora crassa *circadian clock PLoS One 2007 2 8 e795 10.1371/journal.pone.0000795 17726525 PMC1949147

[B13] Cruz F Roux J Robinson-Rechavi M The expansion of amino-acid repeats is not associated to adaptive evolution in mammalian genes BMC Genomics 2009 10 619 10.1186/1471-2164-10-619 20021652 PMC2806350

[B14] Zhang L Yu S Cao Y Wang J Zuo K Qin J Tang K Distributional gradient of amino acid repeats in plant proteins Genome 2006 49 8 900 905 10.1139/G06-054 17036065

[B15] Hancock JM Simon M Simple sequence repeats in proteins and their significance for network evolution Gene 2005 345 1 113 118 10.1016/j.gene.2004.11.023 15716087

[B16] Alba MM Guigo R Comparative analysis of amino acid repeats in rodents and humans Genome Res 2004 14 4 549 554 10.1101/gr.1925704 15059995 PMC383298

[B17] Faux NG Huttley GA Mahmood K Webb GI de la Banda MG Whisstock JC RCPdb: An evolutionary classification and codon usage database for repeat-containing proteins Genome Res 2007 17 7 1118 1127 10.1101/gr.6255407 17567984 PMC1899123

[B18] DePristo MA Zilversmit MM Hartl DL On the abundance, amino acid composition, and evolutionary dynamics of low-complexity regions in proteins Gene 2006 378 19 30 16806741 10.1016/j.gene.2006.03.023

[B19] Mularoni L Ledda A Toll-Riera M Alba MM Natural selection drives the accumulation of amino acid tandem repeats in human proteins Genome Res 2010 20 6 745 754 10.1101/gr.101261.109 20335526 PMC2877571

[B20] Yu F Sabeti PC Hardenbol P Fu Q Fry B Lu X Ghose S Vega R Perez A Pasternak S Positive selection of a pre-expansion CAG repeat of the human SCA2 gene PLoS Genet 2005 1 3 e41 10.1371/journal.pgen.0010041 16205789 PMC1239938

[B21] Hammock EA Young LJ Microsatellite instability generates diversity in brain and sociobehavioral traits Science 2005 308 5728 1630 1634 10.1126/science.1111427 15947188

[B22] Huntley MA Clark AG Evolutionary analysis of amino acid repeats across the genomes of 12 Drosophila species Mol Biol Evol 2007 24 12 2598 2609 10.1093/molbev/msm129 17602168

[B23] Caburet S Vaiman D Veitia RA A genomic basis for the evolution of vertebrate transcription factors containing amino Acid runs Genetics 2004 167 4 1813 1820 10.1534/genetics.104.029082 15342519 PMC1470981

[B24] Maia LC Souza VQ Kopp MM Carvalho FIF Oliveira AC Tandem repeat distribution of gene transcripts in three plant families Genet Mol Biol 2009 32 4 1 12 doi.org/10.1590/S1415-47572009005000091 21637460 10.1590/S1415-47572009005000091 PMC3036893

[B25] Swarbreck D Wilks C Lamesch P Berardini TZ Garcia-Hernandez M Foerster H Li D Meyer T Muller R Ploetz L The Arabidopsis Information Resource (TAIR): gene structure and function annotation Nucleic Acids Res 2008 36 Database D1009 D1014 10.1093/nar/gkm965 PMC2238962 17986450

[B26] Ming R Hou S Feng Y Yu Q Dionne-Laporte A Saw JH Senin P Wang W Ly BV Lewis KL The draft genome of the transgenic tropical fruit tree papaya (*Carica papaya *Linnaeus) Nature 2008 452 7190 991 996 10.1038/nature06856 18432245 PMC2836516

[B27] Schmutz J Cannon SB Schlueter J Ma J Mitros T Nelson W Hyten DL Song Q Thelen JJ Cheng J Genome sequence of the palaeopolyploid soybean Nature 2010 463 7278 178 183 10.1038/nature08670 20075913

[B28] Velasco R Zharkikh A Affourtit J Dhingra A Cestaro A Kalyanaraman A Fontana P Bhatnagar SK Troggio M Pruss D The genome of the domesticated apple (*Malus × domestica *Borkh.) Nat Genet 2010 42 10 833 839 10.1038/ng.654 20802477

[B29] Tuskan GA Difazio S Jansson S Bohlmann J Grigoriev I Hellsten U Putnam N Ralph S Rombauts S Salamov A The genome of black cottonwood, *Populus trichocarpa *(Torr. & Gray) Science 2006 313 5793 1596 1604 10.1126/science.1128691 16973872

[B30] Jaillon O Aury JM Noel B Policriti A Clepet C Casagrande A Choisne N Aubourg S Vitulo N Jubin C The grapevine genome sequence suggests ancestral hexaploidization in major angiosperm phyla Nature 2007 449 7161 463 467 10.1038/nature06148 17721507

[B31] Ouyang S Zhu W Hamilton J Lin H Campbell M Childs K Thibaud-Nissen F Malek RL Lee Y Zheng L The TIGR Rice Genome Annotation Resource: improvements and new features Nucleic Acids Res 2007 35 Database D883 D887 17145706 10.1093/nar/gkl976 PMC1751532

[B32] Vogel JP Garvin DF Mockler TC Schmutz J Rokhsar D Bevan MW Barry K Lucas S Harmon-Smith M Lail K Genome sequencing and analysis of the model grass *Brachypodium distachyon* Nature 2010 463 7282 763 768 10.1038/nature08747 20148030

[B33] Paterson AH Bowers JE Bruggmann R Dubchak I Grimwood J Gundlach H Haberer G Hellsten U Mitros T Poliakov A The *Sorghum bicolor *genome and the diversification of grasses Nature 2009 457 7229 551 556 10.1038/nature07723 19189423

[B34] Schnable PS Ware D Fulton RS Stein JC Wei F Pasternak S Liang C Zhang J Fulton L Graves TA The B73 maize genome: complexity, diversity, and dynamics Science 2009 326 5956 1112 1115 10.1126/science.1178534 19965430

[B35] Rensing SA Lang D Zimmer AD Terry A Salamov A Shapiro H Nishiyama T Perroud PF Lindquist EA Kamisugi Y The *Physcomitrella *genome reveals evolutionary insights into the conquest of land by plants Science 2008 319 5859 64 69 10.1126/science.1150646 18079367

[B36] Merchant SS Prochnik SE Vallon O Harris EH Karpowicz SJ Witman GB Terry A Salamov A Fritz-Laylin LK Marechal-Drouard L The *Chlamydomonas *genome reveals the evolution of key animal and plant functions Science 2007 318 5848 245 250 10.1126/science.1143609 17932292 PMC2875087

[B37] Prochnik SE Umen J Nedelcu AM Hallmann A Miller SM Nishii I Ferris P Kuo A Mitros T Fritz-Laylin LK Genomic analysis of organismal complexity in the multicellular green alga *Volvox carteri* Science 2010 329 5988 223 226 10.1126/science.1188800 20616280 PMC2993248

[B38] Nakachi Y Hayakawa T Oota H Sumiyama K Wang L Ueda S Nucleotide compositional constraints on genomes generate alanine-, glycine-, and proline-rich structures in transcription factors Mol Biol Evol 1997 14 10 1042 1049 9335144 10.1093/oxfordjournals.molbev.a025710

[B39] Meunier J Duret L Recombination drives the evolution of GC-content in the human genome Mol Biol Evol 2004 21 6 984 990 10.1093/molbev/msh070 14963104

[B40] Gaut BS Wright SI Rizzon C Dvorak J Anderson LK Recombination: an underappreciated factor in the evolution of plant genomes Nat Rev Genet 2007 8 1 77 84 10.1038/nrg1970 17173059

[B41] Kim S Plagnol V Hu TT Toomajian C Clark RM Ossowski S Ecker JR Weigel D Nordborg M Recombination and linkage disequilibrium in *Arabidopsis thaliana* Nat Genet 2007 39 9 1151 1155 10.1038/ng2115 17676040

[B42] Zilversmit MM Volkman SK DePristo MA Wirth DF Awadalla P Hartl DL Low-complexity regions in *Plasmodium falciparum*: missing links in the evolution of an extreme genome Mol Biol Evol 2010 27 9 2198 2209 10.1093/molbev/msq108 20427419 PMC2922621

[B43] Hancock JM Worthey EA Santibanez-Koref MF A role for selection in regulating the evolutionary emergence of disease-causing and other coding CAG repeats in humans and mice Mol Biol Evol 2001 18 6 1014 1023 10.1093/oxfordjournals.molbev.a003873 11371590

[B44] Dalby AR A comparative proteomic analysis of the simple amino acid repeat distributions in *Plasmodia *reveals lineage specific amino acid selection PLoS One 2009 4 7 e6231 10.1371/journal.pone.0006231 19597555 PMC2705789

[B45] Monke G Altschmied L Tewes A Reidt W Mock HP Baumlein H Conrad U Seed-specific transcription factors ABI3 and FUS3: molecular interaction with DNA Planta 2004 219 1 158 166 10.1007/s00425-004-1206-9 14767767

[B46] Franks RG Wang C Levin JZ Liu Z SEUSS, a member of a novel family of plant regulatory proteins, represses floral homeotic gene expression with LEUNIG Development 2002 129 1 253 263 11782418 10.1242/dev.129.1.253

[B47] Conner J Liu Z LEUNIG, a putative transcriptional corepressor that regulates AGAMOUS expression during flower development Proc Natl Acad Sci USA 2000 97 23 12902 12907 10.1073/pnas.230352397 11058164 PMC18862

[B48] Cvrckova F Novotny M Pickova D Zarsky V Formin homology 2 domains occur in multiple contexts in angiosperms BMC Genomics 2004 5 1 44 10.1186/1471-2164-5-44 15256004 PMC509240

[B49] Cerdan PD Chory J Regulation of flowering time by light quality Nature 2003 423 6942 881 885 10.1038/nature01636 12815435

[B50] Tompa P Intrinsically unstructured proteins evolve by repeat expansion Bioessays 2003 25 9 847 855 10.1002/bies.10324 12938174

[B51] Williams RM Obradovi Z Mathura V Braun W Garner EC Young J Takayama S Brown CJ Dunker AK The protein non-folding problem: amino acid determinants of intrinsic order and disorder Pac Symp Biocomput 2001 89 100 11262981 10.1142/9789814447362_0010

[B52] Peng K Radivojac P Vucetic S Dunker AK Obradovic Z Length-dependent prediction of protein intrinsic disorder BMC Bioinformatics 2006 7 208 10.1186/1471-2105-7-208 16618368 PMC1479845

[B53] Dosztanyi Z Csizmok V Tompa P Simon I IUPred: web server for the prediction of intrinsically unstructured regions of proteins based on estimated energy content Bioinformatics 2005 21 16 3433 3434 10.1093/bioinformatics/bti541 15955779

[B54] Hirose S Shimizu K Kanai S Kuroda Y Noguchi T POODLE-L: a two-level SVM prediction system for reliably predicting long disordered regions Bioinformatics 2007 23 16 2046 2053 10.1093/bioinformatics/btm302 17545177

[B55] Vavouri T Semple JI Garcia-Verdugo R Lehner B Intrinsic protein disorder and interaction promiscuity are widely associated with dosage sensitivity Cell 2009 138 1 198 208 10.1016/j.cell.2009.04.029 19596244

[B56] Edwards YJ Lobley AE Pentony MM Jones DT Insights into the regulation of intrinsically disordered proteins in the human proteome by analyzing sequence and gene expression data Genome Biol 2009 10 5 R50 10.1186/gb-2009-10-5-r50 19432952 PMC2718516

[B57] Haerty W Golding GB Genome-wide evidence for selection acting on single amino acid repeats Genome Res 2010 20 6 755 760 10.1101/gr.101246.109 20056893 PMC2877572

[B58] TAIR http://www.arabidopsis.org

[B59] Phytozome http://www.phytozome.net

[B60] Ostlund G Schmitt T Forslund K Kostler T Messina DN Roopra S Frings O Sonnhammer EL InParanoid 7: new algorithms and tools for eukaryotic orthology analysis Nucleic Acids Res 2010 38 Database D196 D203 19892828 10.1093/nar/gkp931 PMC2808972

[B61] Team R Development Core R: A language and environment for statistical computing http://www.r-project.org

[B62] Barrett JC Fry B Maller J Daly MJ Haploview: analysis and visualization of LD and haplotype maps Bioinformatics 2005 21 2 263 265 10.1093/bioinformatics/bth457 15297300

[B63] Li N Stephens M Modeling linkage disequilibrium and identifying recombination hotspots using single-nucleotide polymorphism data Genetics 2003 165 4 2213 2233 14704198 10.1093/genetics/165.4.2213 PMC1462870

[B64] Katoh K Toh H Recent developments in the MAFFT multiple sequence alignment program Brief Bioinform 2008 9 4 286 298 10.1093/bib/bbn013 18372315

[B65] Yang Z PAML 4: phylogenetic analysis by maximum likelihood Mol Biol Evol 2007 24 8 1586 1591 10.1093/molbev/msm088 17483113

[B66] Schmid M Davison TS Henz SR Pape UJ Demar M Vingron M Scholkopf B Weigel D Lohmann JU A gene expression map of *Arabidopsis thalian*a development Nat Genet 2005 37 5 501 506 10.1038/ng1543 15806101

[B67] Barrett T Edgar R Gene expression omnibus: microarray data storage, submission, retrieval, and analysis Methods Enzymol 2006 411 352 369 16939800 10.1016/S0076-6879(06)11019-8 PMC1619900

[B68] Fujita M Horiuchi Y Ueda Y Mizuta Y Kubo T Yano K Yamaki S Tsuda K Nagata T Niihama M Rice expression atlas in reproductive development Plant Cell Physiol 2010 51 12 2060 2081 10.1093/pcp/pcq165 21062870

[B69] Jung KH Dardick C Bartley LE Cao P Phetsom J Canlas P Seo YS Shultz M Ouyang S Yuan Q Refinement of light-responsive transcript lists using rice oligonucleotide arrays: evaluation of gene-redundancy PLoS One 2008 3 10 e3337 10.1371/journal.pone.0003337 18836531 PMC2556097

[B70] Li SW Feng L Niu DK Selection for the miniaturization of highly expressed genes Biochem Biophys Res Commun 2007 360 3 586 592 10.1016/j.bbrc.2007.06.085 17610841

[B71] Yanai I Benjamin H Shmoish M Chalifa-Caspi V Shklar M Ophir R Bar-Even A Horn-Saban S Safran M Domany E Genome-wide midrange transcription profiles reveal expression level relationships in human tissue specification Bioinformatics 2005 21 5 650 659 10.1093/bioinformatics/bti042 15388519

[B72] Narsai R Howell KA Millar AH O'Toole N Small I Whelan J Genome-wide analysis of mRNA decay rates and their determinants in *Arabidopsis thaliana* Plant Cell 2007 19 11 3418 3436 10.1105/tpc.107.055046 18024567 PMC2174890

