# Supplementary material for: Comprehensive analysis of tandem amino acid repeats from ten angiosperm genomes
Source: BMC Genomics. 2011 Dec 23;12:632. doi: 10.1186/1471-2164-12-632 (PMC3283746; doi:10.1186/1471-2164-12-632)
Supplement: Additional file 1 — The relationship between AAR content and GC content in fungi and insects. This file contains two tables (Tables S1 and S2) that list the sources of the fungi and insect genome sequences that were used in this study, as well as two figures (Figures S1 and S2) that show the corresponding relationship between AAR content and GC content. [file 1471-2164-12-632-S1.PDF]

Table S1 Summary of eight *Sordariomycetes* fungus genomes under survey

Organism	Source	Version	Reference
Neurospora crassa	FGI [1]	10	[2]
Podospora anserina	PAGP [3]	6.28	[4]
Magnaporthe grisea	FGI [1]	2.3	[5]
Fusarium graminearum	FGI [1]	3	[6]
Fusarium oxysporum	FGI [1]	2	[7]
Fusarium verticillioides	FGI [1]	3	[7]
Trichoderma reesei	JGI [8]	2.0	[9]
Sordaria macrospora	SMGH [10]	2.0	[11]

Table S2 Summary of 15 *Diptera* insect genomes under survey

Organism	Source	Version	Reference
**Fruit flies**			
Drosophila melanogaster	FlyBase [12]	5.29	[13, 14]
Drosophila ananassae	FlyBase [12]	1.3	[14]
Drosophila erecta	FlyBase [12]	1.3	[14]
Drosophila grimshawi	FlyBase [12]	1.3	[14]
Drosophila mojavensis	FlyBase [12]	1.3	[14]
Drosophila persimilis	FlyBase [12]	1.3	[14]
Drosophila pseudoobscura	FlyBase [12]	2.9	[14, 15]
Drosophila sechellia	FlyBase [12]	1.3	[14]
Drosophila simulans	FlyBase [12]	1.3	[14]
Drosophila virilis	FlyBase [12]	1.2	[14]
Drosophila willistoni	FlyBase [12]	1.3	[14]
Drosophila yakuba	FlyBase [12]	1.3	[14]
**Mosquitoes**			
Aedes aegypti	VectorBase [16]	1.2	[17]
Anopheles gambiae	VectorBase [16]	3.6	[18]
Culex quinquefasciatus	VectorBase [16]	1.2	[19]

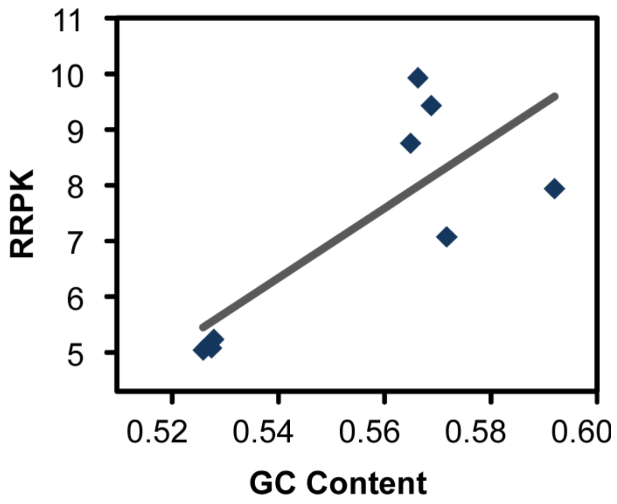

Figure S1 Positive correlation between AAR content and GC content in orthologs among *Sordariomycetes* fungi

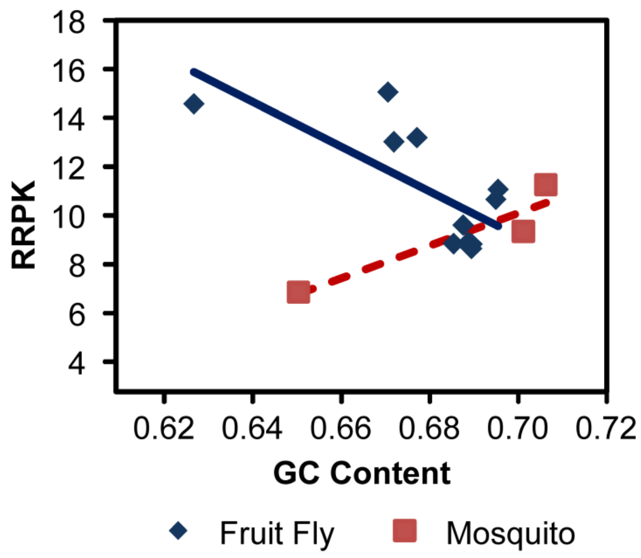

Figure S2 Relationship between AAR content and GC content in orthologs among *Diptera* insects

Negative correlation observed in fruit flies was indicated by blue solid line, while susceptible positive correlation observed in mosquitoes was illustrated as red dash line.

References

1. **Fungal Genome Initiative. [http://www.broadinstitute.org/annotation/fungi]**.

2. Galagan JE, Calvo SE, Borkovich KA, Selker EU, Read ND, Jaffe D, FitzHugh W, Ma LJ, Smirnov S, Purcell S *et al*: **The genome sequence of the filamentous fungus *Neurospora crassa***. *Nature* 2003, **422**(6934):859-868.

3. ***Podospora anserina* Genome Project. [http://podospora.igmors.u-psud.fr]**.

4. Espagne E, Lespinet O, Malagnac F, Da SC, Jaillon O, Porcel BM, Couloux A, Aury JM, Segurens B, Poulain J *et al*: **The genome sequence of the model ascomycete fungus *Podospora anserina***. *Genome Biol* 2008, **9**(5):R77.

5. Dean RA, Talbot NJ, Ebbole DJ, Farman ML, Mitchell TK, Orbach MJ, Thon M, Kulkarni R, Xu JR, Pan H *et al*: **The genome sequence of the rice blast fungus *Magnaporthe grisea***. *Nature* 2005, **434**(7036):980-986.

6. Cuomo CA, Guldener U, Xu JR, Trail F, Turgeon BG, Di Pietro A, Walton JD, Ma LJ, Baker SE, Rep M *et al*: **The *Fusarium graminearum* genome reveals a link between localized polymorphism and pathogen specialization**. *Science* 2007, **317**(5843):1400-1402.

7. Ma LJ, van der Does HC, Borkovich KA, Coleman JJ, Daboussi MJ, Di Pietro A, Dufresne M, Freitag M, Grabherr M, Henrissat B *et al*: **Comparative genomics reveals mobile pathogenicity chromosomes in *Fusarium***. *Nature* 2010, **464**(7287):367-373.

8. **JGI Genome Portal. [http://genome.jgi-psf.org]**.

9. Martinez D, Berka RM, Henrissat B, Saloheimo M, Arvas M, Baker SE, Chapman J, Chertkov O, Coutinho PM, Cullen D *et al*: **Genome sequencing and analysis of the biomass-degrading fungus *Trichoderma reesei* (syn. *Hypocrea jecorina*)**. *Nat Biotechnol* 2008, **26**(5):553-560.

10. **The *Sordaria macrospora* genome homepage at the Ruhr-University Bochum. [http://c4-1-8.serverhosting.rub.de/public/]**.

11. Nowrousian M, Stajich JE, Chu M, Engh I, Espagne E, Halliday K, Kamerewerd J, Kempken F, Knab B, Kuo HC *et al*: **De novo assembly of a 40 Mb eukaryotic genome from short sequence reads: *Sordaria macrospora*, a model organism for fungal morphogenesis**. *PLoS Genet* 2010, **6**(4):e1000891.

12. **FlyBase [http://flybase.org]**.

13. Celniker SE, Wheeler DA, Kronmiller B, Carlson JW, Halpern A, Patel S, Adams M, Champe M, Dugan SP, Frise E *et al*: **Finishing a whole-genome shotgun: release 3 of the *Drosophila melanogaster* euchromatic genome sequence**. *Genome Biol* 2002, **3**(12):H79.

14. Clark AG, Eisen MB, Smith DR, Bergman CM, Oliver B, Markow TA, Kaufman TC, Kellis M, Gelbart W, Iyer VN *et al*: **Evolution of genes and genomes on the *Drosophila* phylogeny**. *Nature* 2007, **450**(7167):203-218.

15. Richards S, Liu Y, Bettencourt BR, Hradecky P, Letovsky S, Nielsen R, Thornton K, Hubisz MJ, Chen R, Meisel RP *et al*: **Comparative genome sequencing of *Drosophila pseudoobscura*: chromosomal, gene, and cis-element evolution**. *Genome Res* 2005, **15**(1):1-18.

16. **VectorBase [http://www.vectorbase.org]**.

17. Nene V, Wortman JR, Lawson D, Haas B, Kodira C, Tu ZJ, Loftus B, Xi Z, Megy K, Grabherr M *et al*: **Genome sequence of *Aedes aegypti*, a major arbovirus vector**. *Science* 2007, **316**(5832):1718-1723.

18. Sharakhova MV, Hammond MP, Lobo NF, Krzywinski J, Unger MF, Hillenmeyer ME, Bruggner RV, Birney E, Collins FH: **Update of the *Anopheles gambiae* PEST genome assembly**. *Genome Biol* 2007, **8**(1):R5.

19. Arensburger P, Megy K, Waterhouse RM, Abrudan J, Amedeo P, Antelo B, Bartholomay L, Bidwell S, Caler E, Camara F *et al*: **Sequencing of *Culex quinquefasciatus* establishes a platform for mosquito comparative genomics**. *Science* 2010, **330**(6000):86-88.
