# Supplementary material for: Comprehensive analysis of tandem amino acid repeats from ten angiosperm genomes
Source: BMC Genomics. 2011 Dec 23;12:632. doi: 10.1186/1471-2164-12-632 (PMC3283746; doi:10.1186/1471-2164-12-632)
Supplement: Additional file 2 — Fast evolving AAR flanking regions, conserved long AARs of unknown function and RRPK of alternatively spliced exons. This file contains Tables S3-S7. [file 1471-2164-12-632-S2.PDF]

Table S3 dN/dS ratio comparison between AAR flanking regions and the whole RCPs

Abbreviation	dN/dS (the whole RCPs)	dN/dS (flanking regions)	p value
Arabidopsis	0.119	0.330	$<2.2\times10^{-16}$
papaya	0.119	0.464	$<2.2\times10^{-16}$
soybean	0.117	0.460	$<2.2\times10^{-16}$
apple	0.128	0.329	$<2.2\times10^{-16}$
cottonwood	0.119	0.503	$<2.2\times10^{-16}$
grape	0.124	0.497	$<2.2\times10^{-16}$
false brome	0.125	0.502	1.87×10^{-10}
rice	0.123	0.356	$<2.2\times10^{-16}$
sorghum	0.124	0.529	7.92×10^{-13}
maize	0.124	0.544	$<2.2\times10^{-16}$

Species abbreviation was described in Table 1. The p value was obtained through Mann-Whitney U test.

Table S4 Fraction of AAR flanking regions with dN/dS larger than 1

Abbreviation	Method	Fraction (%)
Arabidopsis	Average dN/dS	2.93
papaya	Average dN/dS	2.80
soybean	Average dN/dS	2.96
apple	Average dN/dS	3.54
cottonwood	Average dN/dS	3.32
grape	Average dN/dS	3.23
false brome	Average dN/dS	3.01
rice	Average dN/dS	3.08
sorghum	Average dN/dS	3.32
maize	Average dN/dS	3.10
Arabidopsis	Pairwise dN/dS vs. papaya	6.52
papaya	Pairwise dN/dS vs. Arabidopsis	6.13
false brome	Pairwise dN/dS vs. rice	3.48
rice	Pairwise dN/dS vs. false brome	4.82
sorghum	Pairwise dN/dS vs. maize	2.91
maize	Pairwise dN/dS vs. sorghum	2.82

Species abbreviation was described in Table 1.

TableS5 List of function unknown conserved long AARs in Arabidopsis

Protein	Type	Gene Name	Domain [a]
AT1G06230.1	S	*GTE4 (GLOBAL TRANSCRIPTION FACTOR GROUP E4)*	PTHR22880
AT1G65440.1	K	*GTB1 (GLOBAL TRANSCRIPTION FACTOR GROUP B1)*	IPR017072
AT2G26140.1	A	*IQD4(IQ DOMAIN4)*	OOD
AT2G32600.1	P	-	PTHR23205
AT3G04460.1	P	*PEX12(PEROXIN-12)*	IPR017375
AT3G05210.1[b]	P	*ERCC1*	PTHR12749
AT3G05470.1	P	-	PTHR23213
AT3G50340.1	S	-	-
AT4G18570.1	P	-	PTHR23213
AT4G38630.1	A	*RPN10(REGULATORY PARTICLE NON-ATPASE 10)*	PTHR10223
AT4G39790.1	P	-	-
AT5G11700.2	G	-	-
AT5G13780.1	H	-	OOD

[a] If an AAR was mapped to a certain domain by InterProScan [1], the INTERPRO [2] ID or the PANTHER [3] ID was given. If AAR was not settled in any domain (if existed) of a protein, it was labeled as OOD (Out of Domain).[b] Functional necessity of region containing this AAR has been suspected [4].

Table S6 Average AAR content of protein segments encoded by different types of exons in all RCPs' transcripts from the proteomes

	Arabidopsis	rice
RRPK (Alternatively Spliced Exons)	20.6	24.1
RRPK(Constitutively Spliced Exons)	9.8	12.8
p value [a]	1.1×10^{-28}	5.1×10^{-47}

[a] The p value was obtained through Welch's t-test.

Table S7 Average AAR content of protein segments encoded by different types of exons in orthologous RCPs' transcripts

	Arabidopsis	rice
RRPK (Alternatively Spliced Exons)	19.5	22.0
RRPK (Constitutively Spliced Exons)	10.6	12.0
p value [a]	7.6×10^{-6}	4.6×10^{-11}

[a] The p value was obtained through Welch's t-test.

References

1. Quevillon E, Silventoinen V, Pillai S, Harte N, Mulder N, Apweiler R, Lopez R: **InterProScan: protein domains identifier**. *Nucleic Acids Res* 2005, **33**(Web Server issue):W116-W120.

2. Hunter S, Apweiler R, Attwood TK, Bairoch A, Bateman A, Binns D, Bork P, Das U, Daugherty L, Duquenne L *et al*: **InterPro: the integrative protein signature database**. *Nucleic Acids Res* 2009, **37**(Database issue):D211-D215.

3. Mi H, Dong Q, Muruganujan A, Gaudet P, Lewis S, Thomas PD: **PANTHER version 7: improved phylogenetic trees, orthologs and collaboration with the Gene Ontology Consortium**. *Nucleic Acids Res* 2010, **38**(Database issue):D204-D210.

4. Tsodikov OV, Enzlin JH, Scharer OD, Ellenberger T: **Crystal structure and DNA binding functions of ERCC1, a subunit of the DNA structure-specific endonuclease XPF-ERCC1**. *Proc Natl Acad Sci U S A* 2005, **102**(32):11236-11241.
